# Supplementary material for: Dermatologist-like explainable AI enhances trust and confidence in diagnosing melanoma
Source: Nat Commun. 2024 Jan 15;15:524. doi: 10.1038/s41467-023-43095-4 (PMC10789736; doi:10.1038/s41467-023-43095-4)
Supplement: Supplementary file 1 — Supplementary Information [file 41467_2023_43095_MOESM1_ESM.pdf]

Supplementary Information

Dermatologist-like explainable AI enhances trust and confidence in diagnosing melanoma

a **b**

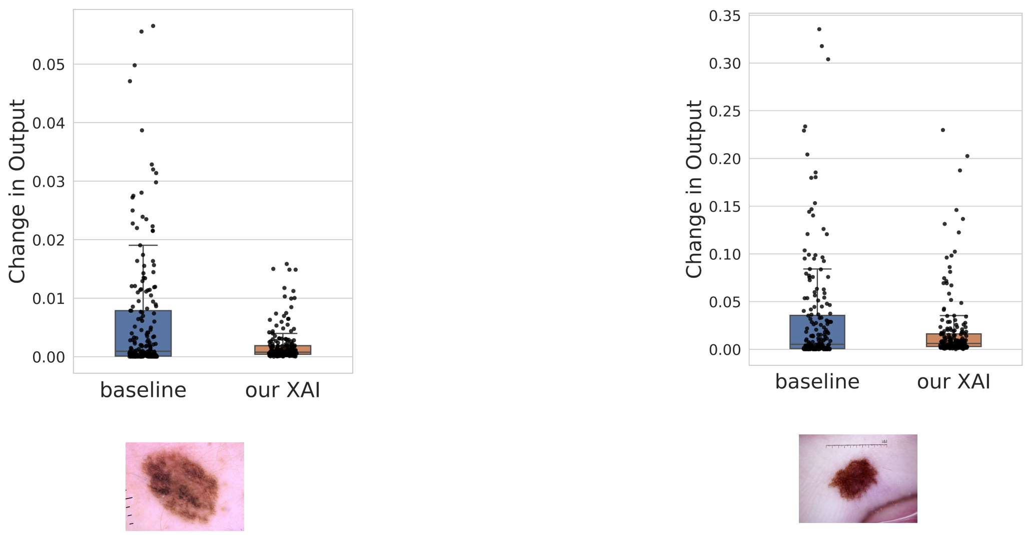

ruler 1 **ruler 2**

Supplementary Fig. 1: Robustness of our XAI to artefacts.

a, **b**: Sensitivity of our XAI and the baseline classifier to the presence of different rulers. We selected two frequently occurring rulers from our training set and superimposed them on each image in the test set. The y-axis represents the absolute change in classifier logit output after superimposing each of two rulers. Since our XAI has multiple output scores, that is, one for each characteristic, we averaged the output scores per lesion to obtain a single value to facilitate a comparison with the baseline. To assess the sensitivity to artefacts, we computed the mean change in the output score of our XAI and of the baseline classifier when superimposing a ruler on the images. For the images on which we superimposed ruler 1, the mean absolute change in the output score was 0.001 (95% CI: [0.001, 0.002]) for our XAI and 0.006 (95% CI: [0.005, 0.008]) for the baseline classifier ($P<0.0001$, two-sided paired t test, n=196 images). For ruler 2, the mean absolute change in the output score for our XAI was 0.018 (95% CI: [0.014, 0.024]), and that for the baseline classifier 0.032 (95% CI: [0.024, 0.041]) ($P=0.005$, two-sided paired t test, n=196 images). The horizontal line on each box denotes the median value and the white dot denotes the mean. The upper and lower box limits denote the 1st and 3rd quartiles, respectively, and the whiskers extend from the box to 1.5 times the interquartile range. Example images are shown of two lesions with superimposed ruler artefacts characterised by dark lines on the images. Source data are provided as a Source Data file.

Architecture	Balanced Accuracy (baseline)	Balanced Accuracy (our XAI)	Number of Parameters	FLOPS
ResNet-18	**78.5%** (72.5%, 84%)	**78.5%** (72.8%, 84.1%)	11.2 M	1823522304
ResNet-34	76.5% (70.5%, 82.2%)	**77%** (71.1%, 82.7%)	21.3 M	3678227968
ResNet-50	80% (74.4%, 85.4%)	**81%** (75.6%, 86.3%)	23.5 M	4131696640
ResNet-101	80% (74.2%, 85.4%)	**80.5%** (74.8%, 85.8%)	42.5M	7864389632
DenseNet-121	**78.5%** (72.6%, 84%)	75.5% (69.4%, 81.4%)	7.0 M	2895984128
DenseNet-161	77.5% (71.5%, 83.1%)	**79%** (73.3%, 84.5%)	26.5 M	7843635264
EfficientNet-B1	72% (65.7%, 78.1%)	**74%** (67.7%, 80%)	6.5 M	37427584
EfficientNet-B3	74% (67.9%, 80%)	**78%** (72.1%, 83.5%)	10.7 M	52023040

Supplementary Tab. 1: Comparison with multiple CNN architectures.

We ablated the backbone architecture of our XAI and the baseline with eight CNN architectures. The table displays the balanced accuracies obtained with each backbone, with the higher accuracy emphasised in bold. The 95% bootstrap confidence intervals are stated in parentheses next to the balanced accuracies. Source data are provided as a Source Data file.

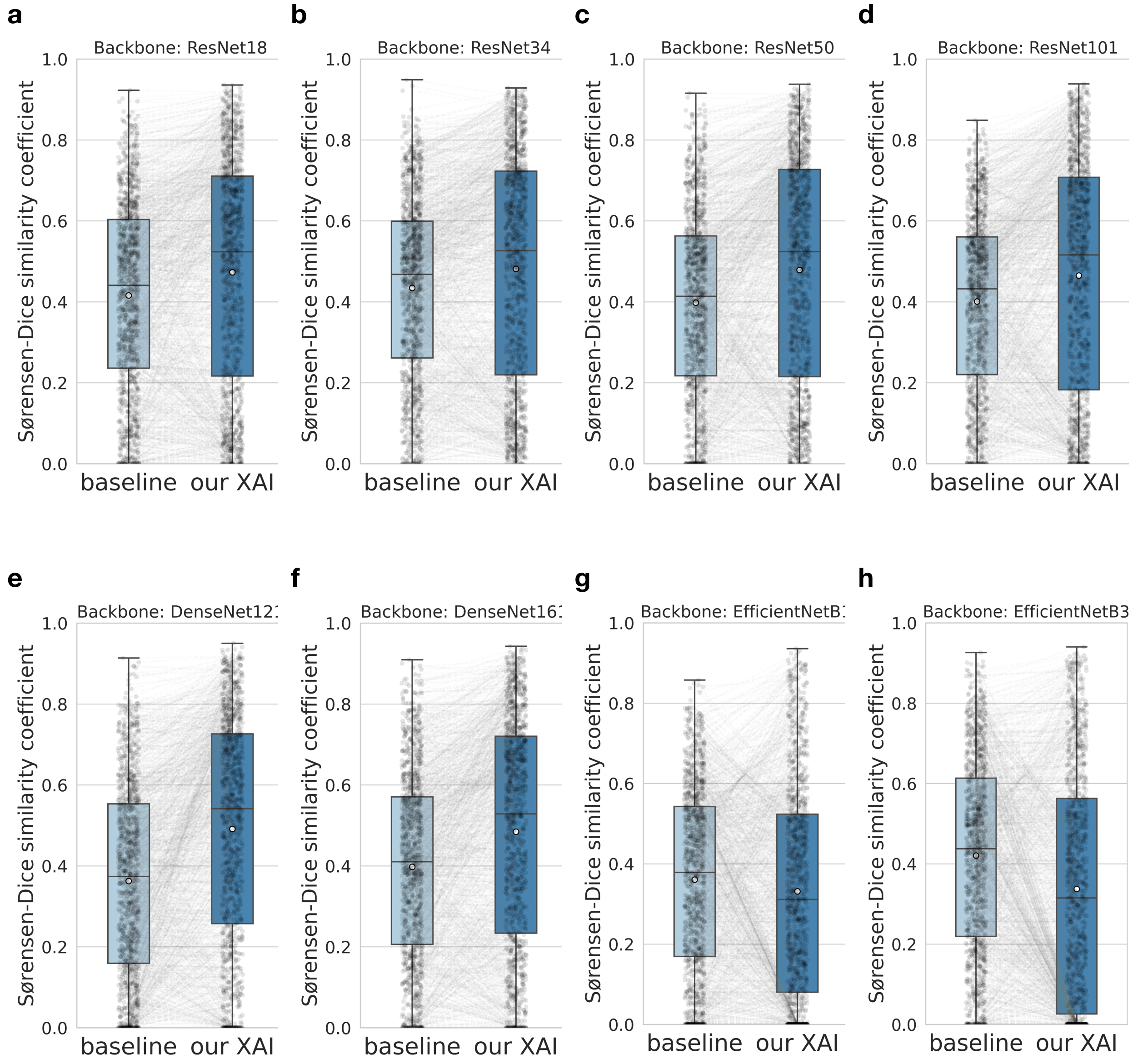

Supplementary Fig. 2: Overlap between human and machine ROIs.

We calculated the ROI overlap for an image as the Sørensen-Dice similarity coefficient (DSC) between human and machine ROIs using ResNet18 **(a)**, ResNet34 **(b)**, ResNet50 **(c)**, ResNet101 **(d)**, DenseNet121 **(e)**, DenseNet161 **(f)**, EfficientNetB1 **(g)**, EfficientNetB3 **(h)**. Each boxplot contains n=1120 images. The horizontal line on each box denotes the median value and the white dot denotes the mean. The upper and lower box limits denote the 1st and 3rd quartiles, respectively, and the whiskers extend from the box to 1.5 times the interquartile range. Source data are provided as a Source Data file.

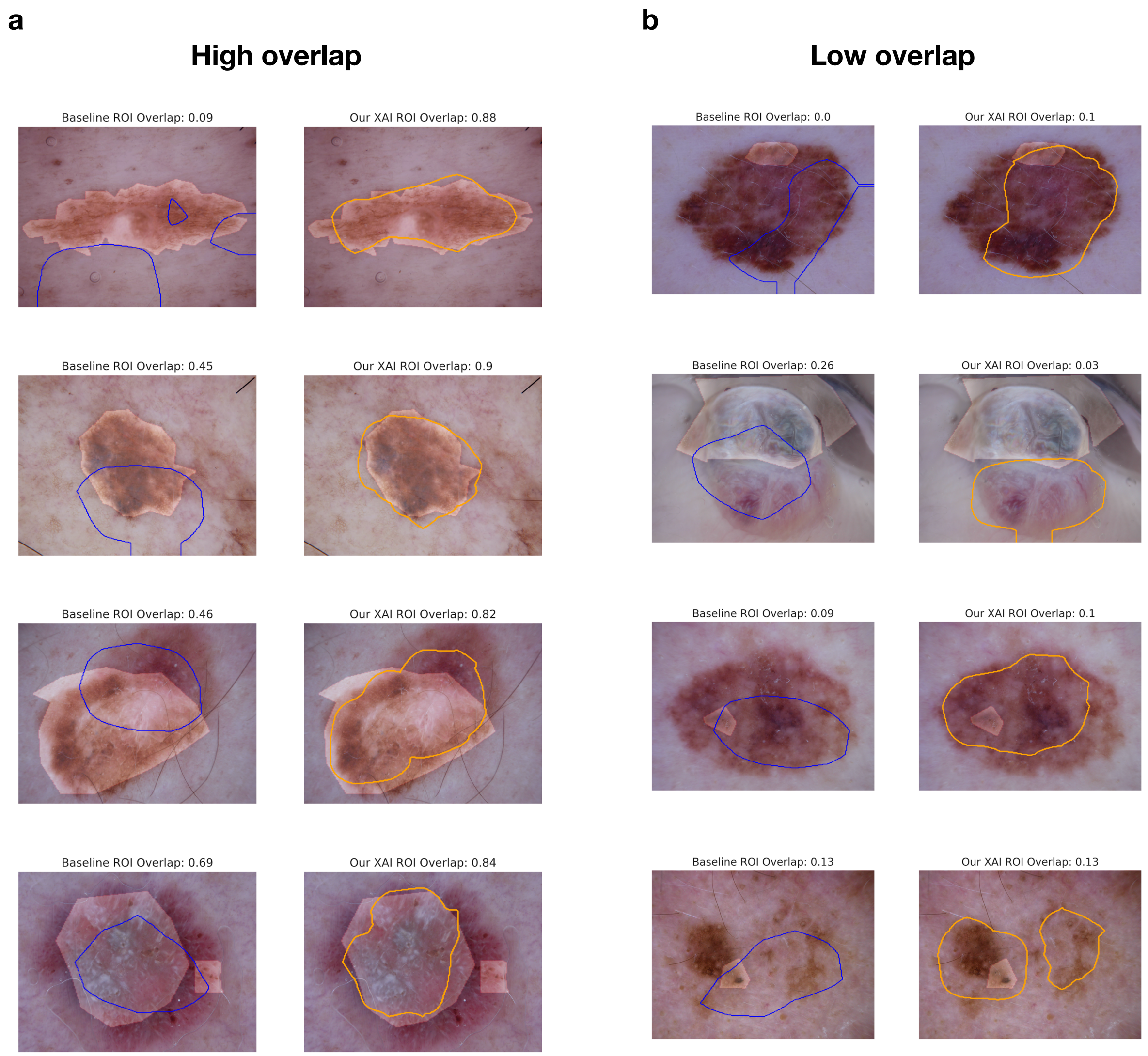

Supplementary Fig. 3: Examples of high and low overlap between clinician and AI regions of interest (ROIs).

a, b: Examples of low (**a**) and high (**b**) overlap between clinicians and the AI. The ROIs of the baseline classifier are marked with blue polygons, and the ROIs of our XAI are marked with orange polygons.

	Sensitivity	Specificity
**Phase 1**	59.55%	72.84%
**Phase 2**	63.86%	80.75%
**Phase 3**	67.86%	78.58%
	**Sensitivity (excision)**	**Specificity (excision)**
**Phase 1**	74.46%	57.64%
**Phase 2**	90.34%	48.82%
**Phase 3**	90.46%	45.97%

Supplementary Tab. 2: Clinician diagnostic performance in all three phases.

The top three rows diagnostic sensitivity and specificity when considering a “nevus excise” diagnosis as a nevus (a melanoma that is wrongly diagnosed as a nevus but treated by excision is considered an incorrect choice). The bottom three rows show excision performance (a melanoma that is wrongly diagnosed as a nevus but treated by excision is considered a correct choice). Source data are provided as a Source Data file.

	Confidence	Trust
**Phase 1**	6.66 (95% CI 6.43, 6.89)	-
**Phase 2**	7.52 (95% CI 7.43, 7.6)	6.72 (95% CI 6.61, 6.83)
**Phase 3**	7.78 (95% CI 7.71, 7.86)	6.96 (95% CI 6.84, 7.1)

Supplementary Tab. 3: Mean confidence and trust at the clinician level in all phases.

Confidence and trust scores were measured on a Likert scale from 1-10, with 1 indicating the lowest confidence/trust and 10 the highest. Source data are provided as a Source Data file.

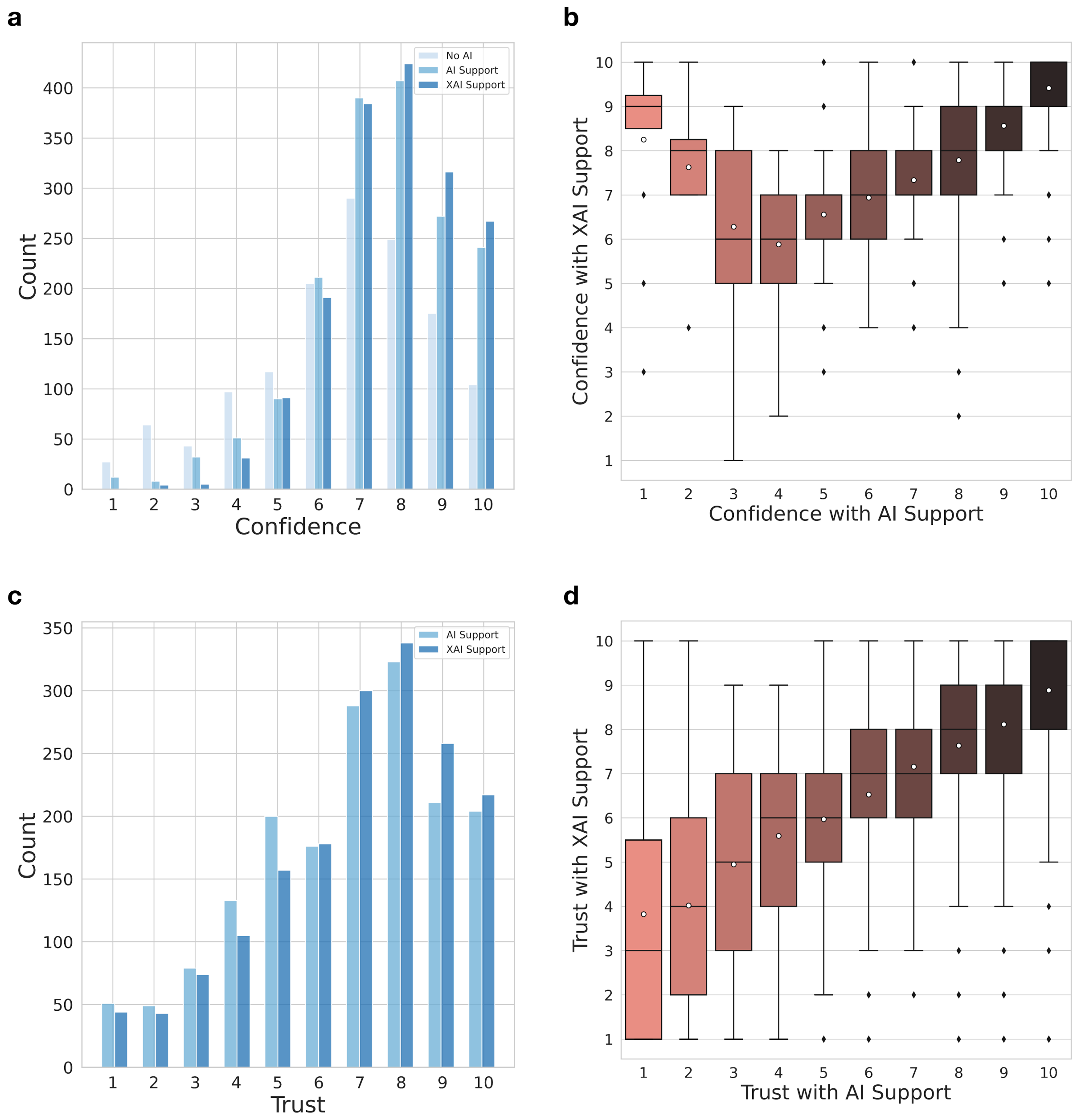

Supplementary Fig. 4: Confidence and trust scores in all phases.

a, c: Histograms of the confidence (**a**) and trust (**c**) values entered by the clinicians for all phases (phase 1: No AI, phase 2: AI support, phase 3: XAI support). Both confidence and trust were measured on a Likert scale from 1-10, with 1 indicating the lowest and 10 the highest confidence/trust. **b, d**: Box plots of the confidence (n=1714 images) (**b**) and trust (n=1714 images) (**d**) values entered by the clinicians for phase 2 (AI support) and phase 3 (XAI support). The figures depict a shift towards higher confidence and trust values as the level of AI support increased. Source data are provided as a Source Data file.

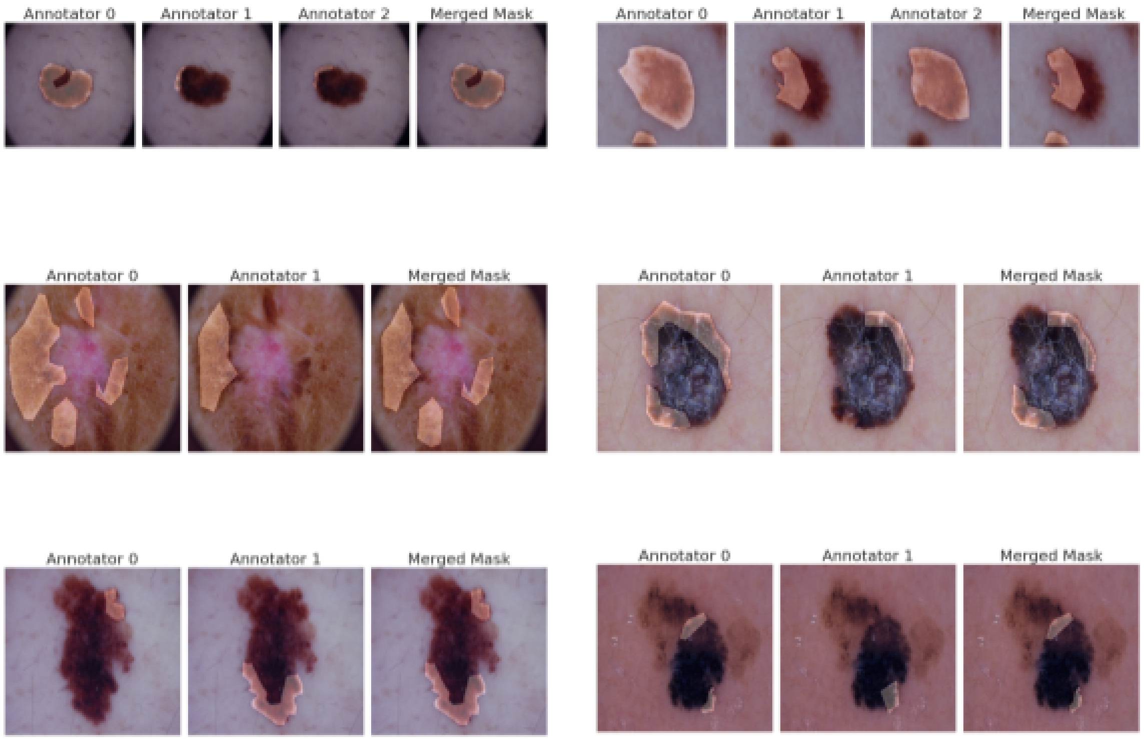

Supplementary Fig. 5: Examples of merged masks.

Examples of merged masks using the chosen merging technique. Valid annotated areas are not lost.

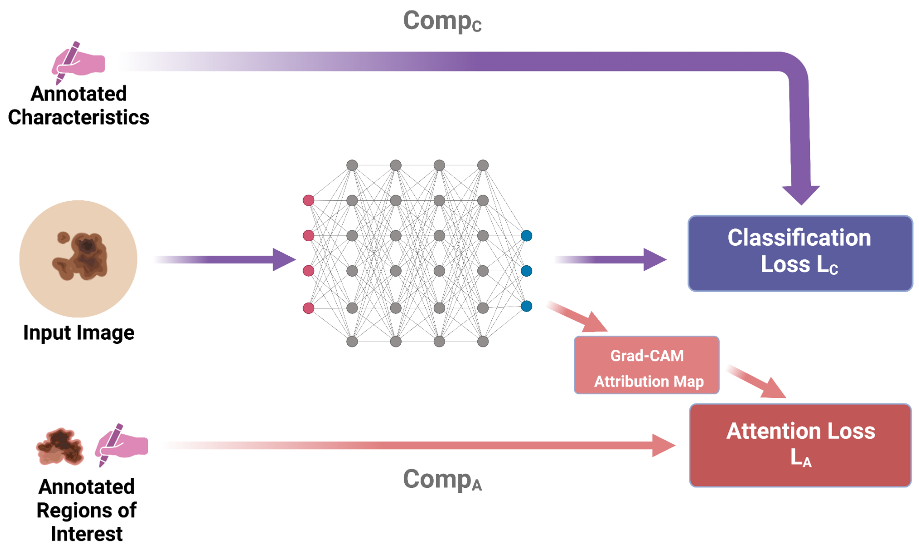

Supplementary Fig. 6: Training setup of our XAI

The input image, the annotated characteristics, and the corresponding regions of interest are used to train the network. $Comp_C$ predicts the characteristics from our ontology. $Comp_A$ computes the loss between the Grad-CAM attributions and the ground truth regions of interest. Figure created with BioRender.com.

Supplementary Information A

Diagnostic accuracy reporting according to the STARD 2015 list (https://bmjopen.bmj.com/content/6/11/e012799).

1. Diagnostic accuracy mentioned in Abstract.
2. Summary provided.
3. Yes. Detailed in the Main section.
4. Yes. Detailed in the Main section.
5. Data collection was planned and performed prior to the index test.
6. Detailed in Methods, subheadings Study Design and Exclusion Criteria.
7. Detailed in Methods, subheading Study Design.
8. Detailed in Methods, subheading Study Design.
9. Sampling strategy detailed in Methods, subheading Study Design.
10. -
11. -
12. -
13. -
14. Comparisons provided in Results.
15. -
16. Missing data was dropped.
17. Subgroup analysis performed.
18. Detailed in Methods under subheading Study Design.
19. Flow of participants detailed in Methods under Study Design.
20. We did not collect demographic information.
21. -
22. Detailed in Methods under subheading Study Design.
23. -
24. Confidence intervals provided for all estimates.
25. -
26. Limitations discussed in the Discussion section.
27. -
28. Study registered on Open Science Framework, linked in Methods.
29. Study registration link provided in Methods.
30. -

Supplementary Information B

Pilot Study

Prior to the three-phase reader study we conducted a pilot study to (1) test the annotation framework with nontechnical users, (2) receive additional feedback on the explanatory ontology and (3) determine how many images we could show the study participants in the targeted processing time of 30 minutes.

For the pilot study we contacted 12 board-certified dermatologists from our network in May 2022 asking them to take 30 minutes to annotate 40 images of melanomas and nevi (19 unique melanomas and nevi, two repeated images) in the time from 20.05.22 to 31.05.22. The pilot dermatologists received a video explaining the use of the annotation framework as well as the annotation process. We conducted feedback rounds between the 01.06.22 and the 07.06.22.

In addition to being able to select a diagnosis, explanations and a confidence value, the pilot study participants could enter additional relevant features or comments in a free text field.

Changes to the Annotation Framework

We identified two common issues regarding the usage of PlainSight in the pilot study. The first revolved around the participants forgetting to select a confidence score or forgetting to save their annotations. We addressed this by explicitly stating the need to do both in an updated version of the explanatory video as well as adding a step-by-step guide to annotations to a text document the participants of the three-phase reader study received by email.

The second issue concerned an issue with the user interface (UI) that made it very difficult for pilot study participants to select explanations. Generally speaking, to interact with objects in a UI, e.g., to open a drop-down menu to select an explanation, users need to interact with parts of the UI, e.g., click into a specific area; usually a box around a button or a line of text. In PlainSight this clickable area is realised as a box around the text of the first entry in the list of possible explanations. As we expected that participants would occasionally forget to choose an explanation, we could not use an actual explanation from the ontology as we wanted to avoid introducing any noise to the data. We therefore chose to simply leave the first entry blank. Unfortunately, this resulted in the clickable area to open the drop-down menu to be a very small box on the left side of the menu. As this is a fairly common issue with UI, it was not obvious to the data scientist setting up the framework (KH) that this would pose a problem to nontechnical users.

As a number of participants specifically asked about this issue, we could address it in these cases in the pilot and thus receive pilot annotations.

We resolved this issue in the three-phase reader study by using a placeholder text (“Please select an explanation.”) instead of an empty field.

Explanatory Ontology

In April 2022 we asked the dermatologists from our network for initial feedback on the ontology and added “thick branched lines” to the melanoma features “Thick reticular or branched lines”. After the pilot study we added the melanoma feature “Pigmentation invades the openings of hair follicles (facial lesions)” as several dermatologists had remarked on this feature in the free text field.

Number of Images

A preliminary test found that 40 images could be annotated within 30 minutes. However, we substantially underestimated the time necessary to familiarise with both the ontology and the framework. As a consequence of the pilot study results, we significantly reduced the number of shown images to 15 to allow the participants of the three-phase reader study enough time for thorough familiarisation with the framework and precise annotation of the lesions in the targeted 30 minute timeframe.

Participation in the Remainder of the Study

We offered the dermatologists who completed the pilot study to participate in the three-phase reader study. For the participants from whom we had received a complete dataset (diagnoses, explanations and confidence values) we used the first 15 images as phase 1 images and contacted them again for phase 2 and 3. The participants from whom we had not received a complete dataset were contacted for phase 1 and participated in the three-phase reader study with 15 images not included in the pilot.

Supplementary Information C

Ontology Creation

To facilitate a speedy explanation for the study participants and ensure a streamlined evaluation on our end, we decided to employ an explanatory ontology instead of free text explanations.

While melanomas are straightforward to explain by the presence of malignant characteristics, nevi can be explained by either only the absence of malignancy (as is implicitly the case in the 7-point checklist, for example) or by both the presence of benign and the absence of malignant patterns. Since the presence of certain characteristics lends itself to explanations that are easy to understand, we decided to use distinct features of both melanomas and nevi with the understanding that the diagnosis of a nevus always implies the absence of melanoma characteristics and that the diagnosis of a melanoma does not rule out the presence of benign patterns.

We collected the criteria of melanomas and nevi presented in the 7-point checklist[1], the derm7pt dataset[2] (excluding criteria that were relevant for the differential diagnosis of melanomas and other non-nevus skin conditions) and "Dermatoscopy"[3]. Supplemental criteria were adopted from the participants of our pilot study and the ground-truth annotators.

For consistency, we exclusively used descriptive terminology in our ontology; however, the participants received two "translations" between our ontology and the 7-point checklist. The "translation" from the descriptive terms used in our ontology to the metaphorical terms used in the 7-point checklist and derm7pt dataset was included in Tables B1 and B2. The "translation" from metaphorical to descriptive terminology can be found in Tables B3 and B4.

The original ontology was compiled in German and translated to English for the international participants. Two board-certified dermatologists (RB, MLV) approved the translation.

Full Ontology with Feature Explanations and "Translations"

The study participants had access to a document containing the following features and explanations. We included the "translation" from descriptive to metaphorical terminology into Tables B1 and B2. Tables B3 and B4 contain the "translation" from metaphorical to descriptive terminology.

Please note that the metaphorical terms used here resulted from a combination of the criteria of the 7-point checklist[1] and the derm7pt dataset[2]. Furthermore, there is no complete agreement between our ontology and the 7-point checklist/derm7pt dataset as we consider a number of

special cases (including acral lesions and recurrences) that are not covered by the 7-point checklist/derm7pt dataset. Additionally, a major difference between our ontology and the 7-point checklist is that “asymmetric combinations of patterns and/or colours” are listed as nevus criteria in our ontology rather than as a melanoma criteria as in the 7-point checklist. The background of this decision is that asymmetry without other melanoma-specific features is not sufficient to establish a diagnosis of melanoma, neither in the context of the 7-point checklist nor in the approach proposed by Kittler and Tschandl[3] (refer to Chapter 5).

The melanoma criteria from our ontology, their explanations as well as the “translation” into metaphorical terminology

Feature: Thick reticular or branched lines
Explanation: Thick lines refer to lines that are at least as wide as the areas between them.
Corresponding feature(s) from the 7-point checklist:

- Atypical pigment network (thick lines)

Feature: Eccentrically located structureless area (any colour except skin colour, white and grey)
Explanation: Relevant for this criterion is the noncentral location of the structureless area. White and grey structureless areas are covered by the melanoma criteria "White lines or white structureless area" and "grey patterns", as well as by the nevus criterion "Melanoma simulator", regardless of their location.
Corresponding feature(s) from the 7-point checklist:

- Irregular blotch(es)
- Blue–white veil (if blue and eccentrically located)

Feature: Grey patterns
Explanation: This criterion refers to grey structureless areas (regardless of their localization) as well as to grey circles, lines, dots or globules. There is overlap between this criterion and the nevus criterion "Melanoma simulator".
Corresponding feature(s) from the 7-point checklist:

- Regression pattern (peppering)
- Atypical pigment network (grey colour)
- Irregular dots and globes (grey)

Feature: Polymorphous vessels
Explanation: This signifies several types of vessels occurring together, for example, dot-like and curved vessels.
Corresponding feature(s) from the 7-point checklist:

- Atypical vascular pattern

Feature: Pseudopods or radial lines at the lesion margin that do not occupy the entire lesional circumference
Explanation: What is relevant here is that the pseudopods/radial lines affect only part of the lesion. The colour of the pattern is irrelevant in this case.
Corresponding feature(s) from the 7-point checklist:

- Irregular streaks

Feature: Black dots or globules in the periphery of the lesion
Explanation: In particular, the dots or globules are not symmetrically arranged with other patterns (for example, reticular lines).
Corresponding feature(s) from the 7-point checklist:

- Irregular dots and globules (grey)

Feature: White lines or white structureless area
Explanation: The localization of the structures is irrelevant for this criterion. There is overlap with the nevus criterion "melanoma simulator".
Corresponding feature(s) from the 7-point checklist:

- Regression pattern (scar-like pigment loss)

Feature: Parallel lines on ridges (acral lesions only)
Explanation: What is relevant here is that the furrows (often only visible at the lesional margin) are not pigmented.
Corresponding feature(s) from the 7-point checklist:

- No corresponding criteria

Feature: Pigmentation extends beyond the area of the scar (only after excision)
Explanation: This refers to cases in which a recurrent melanoma grows beyond the margin of the excised area.
Corresponding feature(s) from the 7-point checklist:

- No corresponding criteria

Feature: Pigmentation invades the openings of hair follicles (facial lesions)
Explanation: Pigmentation of advanced melanomas on the face may involve the openings of hair follicles.
Corresponding feature(s) from the 7-point checklist:

- No corresponding criteria

The nevus criteria from our ontology, their explanations as well as the “translation” into metaphorical terminology.

Feature: Only one pattern and only one colour
Explanation: For example, only brown reticular lines or only skin-coloured globules. This would also include a blue nevus that presents only as blue and structureless.
Corresponding feature(s) from the 7-point checklist:

- Typical pigment network

Feature: Symmetrical combination of patterns and/or colours
Explanation: Two types of symmetric combinations are possible: 1. A pattern and/or colour is evenly distributed within another pattern and/or colour (e.g., dark brown dots on light brown reticular lines). 2. A pattern and/or colour is centrally located within another pattern and/or colour (dark brown reticular lines within light brown reticular lines, dark brown/black globules within dark brown/black radial lines, light brown structureless area centrally within dark brown reticular lines). Melanoma simulators may also show symmetrical combinations, e.g., grey–white central structureless area within skin-coloured globules in the case of a nonpigmented Spitz nevus).
Corresponding feature(s) from the 7-point checklist:

- Regular ‘blotch(es)’
- Regular dots and globules

Feature: Monomorphic vascular pattern
Explanation: Only one type of vessel is present.
Corresponding feature(s) from the 7-point checklist:

- Typical vascular pattern

Feature: Pseudopods or radial lines at the lesional margin involving the entire lesional circumference
Explanation: Strictly speaking, this criterion is a special case of a symmetrical pattern combination. It is listed separately here to distinguish it from the melanoma criterion "Pseudopods or radial lines at the lesional margin that do not involve the entire lesional circumference".
Corresponding feature(s) from the 7-point checklist:

- Regular streaks

Feature: Parallel lines in the furrows (acral lesions only)
Explanation: In acral nevi, pigment may also be located on the ridges; in this case, parallel lines in the furrows are found mainly at the lesional margin.
Corresponding feature(s) from the 7-point checklist:

- No corresponding criteria

Feature: Pigmentation does not extend beyond the area of the scar (only after excision)
Explanation: Recurrent nevi typically do not extend beyond the scar area.
Corresponding feature(s) from the 7-point checklist:
- No corresponding criteria

Feature: Asymmetric combination of multiple patterns and/or colours in the absence of other melanoma criteria
Explanation: Despite the asymmetry, no other clear-cut melanoma criteria are present; thus, the lesion can in principle be evaluated as benign.
Corresponding feature(s) from the 7-point checklist:
- Atypical pigment network (high variability in colour, spacing and thickness of lines, asymmetrically hyperpigmented).
- Irregular dots and globules (except grey and black)

Feature: Melanoma simulator
Explanation: This criterion is used to distinguish melanomas from nevus types that can (and in the present cases do) exhibit melanoma features. Relevant features are, e.g., white lines or grey areas in blue, Reed and Spitz nevi. The underlying idea is that the lesion in this particular case is in principle considered benign despite the appearance of melanoma criteria.
Corresponding feature(s) from the 7-point checklist:
- No corresponding criteria

The melanoma criteria from the 7-point checklist and their "translation" into the descriptive terms used in our ontology.

Feature: Atypical pigment network
Corresponding feature(s) from our ontology:
- Thick reticular or branched lines
- Grey patterns
- Asymmetric combination of multiple patterns and/or colours without other melanoma criteria

Feature: Blue and white veil
Corresponding feature(s) from our ontology:
- Eccentrically located structureless area (any colour except skin colour, white and grey)
- White lines or white structureless area
- Melanoma simulator (if blue nevus with white pattern - nevus criterion)

- Only one pattern and only one colour (if blue nevus without white pattern - nevus criterion)

Feature: Atypical vascular pattern
Corresponding feature(s) from our ontology:
- Polymorphous vessels

Feature: Irregular streaks
Corresponding feature(s) from our ontology:
- Pseudopods or radial lines at the lesion margin that do not occupy the entire lesion circumference

Feature: Irregular "blotch(es)"
Corresponding feature(s) from our ontology:
- Eccentrically located structureless area (any colour except skin colour, white and grey)
- Asymmetric combination of multiple patterns and/or colours without other melanoma criteria (nevus criterion)

Feature: Irregular dots and globules
Corresponding feature(s) from our ontology:
- Grey patterns
- Black dots or globules in the periphery of the lesion
- Asymmetric combination of multiple patterns and/or colours without other melanoma criteria (nevus criterion)

The nevus criteria from the 7-point checklist and derm7pt dataset and their “translation” into the descriptive terms used in our ontology.

Feature: Typical pigment network
Corresponding feature(s) from our ontology:
- Symmetrical combination of patterns and/or colours
- Only one pattern <u>and</u> only one colour

Feature: Typical vascular pattern
Corresponding feature(s) from our ontology:
- Monomorphic vascular pattern

Feature: Regular streaks
Corresponding feature(s) from our ontology:
- Pseudopods or radial lines at the lesion margin over the entire lesion circumference

Feature: Regular 'blotch'
Corresponding feature(s) from our ontology:
- Symmetrical combination of patterns and/or colours

Feature: Regular dots and globules
Corresponding feature(s) from our ontology:
- Symmetrical combination of patterns and/or colours

Supplementary Information D

Merging Ground-Truth Annotations

Since we used at least two annotators per image, we needed to develop a strategy to merge both textual and region of interest (ROI) annotations.

The first step was to decide how we were going to merge the textual explanations. As an example, let us assume that Annotator 1 selects the characteristics [BDG, ESA, TRBL] (BDG = black dots or globules in the periphery of the lesion, ESA = eccentrically located structureless area, TRBL = thick reticular or branched lines). Annotator 2 selects [BDG, GP] (GP = grey patterns). We first considered an intersection approach that would derive the final label as [BDG]. However, we noticed that Annotator 1 selected many explanations (mean: 2.96$\pm$1.24), while certain others selected fewer (1.21$\pm$0.7). Since different annotators focused on and made use of different, but valid, characteristics, we decided to use a union approach instead. Therefore, the resulting explanations for the example above would be [BDG, ESA, TRBL, GP]. Hence, by combining both of their selected explanations, we obtain a more well-rounded dataset to feed into the classifier.

Next, we needed to decide on a strategy for combining the ROIs between multiple annotators for the same explanation. The most straightforward approach to address this from a statistics perspective would have been to apply the STAPLE algorithm. However, we had too few annotators to properly estimate rater accuracy[4], which is a core part of the algorithm, and thus, opted for a different approach.

The next option was to perform an intersection, i.e., the final ROI would have consisted of only the shared pixels of the annotations. However, this approach presented some challenges. Valid annotated regions were lost when merging using an intersection because the regions are either discontinuous or substantially different in size. Intuitively, this suggests that one annotator sees a certain feature on one part of the lesion but fails to notice that it is also present in other parts of the lesion. Additionally, we found variations in the annotations of the same image regions due to distinct but valid annotation styles. By performing an intersection, we would have ended up with the annotations of the annotator who drew the smallest areas. Therefore, merging annotations using an intersection approach did not work for us either.

We circumvented this problem by attempting to measure the amount of information lost when intersecting two annotations and then finding a balance between information and noise. We did this by first intersecting the annotations for an image. We then took each individual annotated polygon and calculated the percentage of pixels lost. We added the polygon back to the intersected annotation if the pixel loss percentage was above a threshold. We defined the pixel loss percentage as *(activate pixels in original polygon - activate pixels in merged annotation*

mask/activate pixels in original polygon). We empirically determined that 0.8 was a suitable threshold. Example merges using this technique are shown in Supplementary Fig. 5. This method of merging provided us with the most suitable annotations for our purpose.

References

1. Argenziano, G. *et al.* Seven-point checklist of dermoscopy revisited. *Br. J. Dermatol.* **164**, 785–790 (2011).
2. Kawahara, J., Daneshvar, S., Argenziano, G. & Hamarneh, G. Seven-Point Checklist and Skin Lesion Classification Using Multitask Multimodal Neural Nets. *IEEE J. Biomed. Health Inform.* **23**, 538–546 (2019).
3. Kittler, Harald, P., Tschandl. *Dermatoskopie*. (Facultas, 2015).
4. Van Leemput, K. & Sabuncu, M. R. A Cautionary Analysis of STAPLE Using Direct Inference of Segmentation Truth. in *Medical Image Computing and Computer-Assisted Intervention – MICCAI 2014* (eds. Golland, P., Hata, N., Barillot, C., Hornegger, J. & Howe, R.) 398–406 (2014). doi:10.1007/978-3-319-10404-1_50.
