# Supplementary material for: Dermatologist-like explainable AI enhances trust and confidence in diagnosing melanoma
Source: Nat Commun. 2024 Jan 15;15:524. doi: 10.1038/s41467-023-43095-4 (PMC10789736; doi:10.1038/s41467-023-43095-4)
Supplement: Supplementary file 3 — Description of Additional Supplementary Files [file 41467_2023_43095_MOESM3_ESM.docx]

**Description of Additional Supplementary Files**

Supplementary Data 1

Description: This file contains the names and affiliations of our reader study participants.

Supplementary Data 2

Description: This file contains the HAM10000 image identifiers of the train and test images used in our work, as indicated by the “split” column. The “group” column indicates the test group the image was allotted to.
