# Supplementary material for: Dermatologist-like explainable AI enhances trust and confidence in diagnosing melanoma
Source: Nat Commun. 2024 Jan 15;15:524. doi: 10.1038/s41467-023-43095-4 (PMC10789736; doi:10.1038/s41467-023-43095-4)
Supplement: Supplementary file 5 — Reporting Summary [file 41467_2023_43095_MOESM5_ESM.pdf]

nature portfolio

Corresponding author(s): Titus J. Brinker

Last updated by author(s): 15/09/23

Reporting Summary

Nature Portfolio wishes to improve the reproducibility of the work that we publish. This form provides structure for consistency and transparency in reporting. For further information on Nature Portfolio policies, see our Editorial Policies and the Editorial Policy Checklist.

Statistics

For all statistical analyses, confirm that the following items are present in the figure legend, table legend, main text, or Methods section.

n/a	Confirmed	
☐	☒	The exact sample size (n) for each experimental group/condition, given as a discrete number and unit of measurement
☐	☒	A statement on whether measurements were taken from distinct samples or whether the same sample was measured repeatedly
☐	☒	The statistical test(s) used AND whether they are one- or two-sided<br>*Only common tests should be described solely by name; describe more complex techniques in the Methods section.*
☐	☒	A description of all covariates tested
☐	☒	A description of any assumptions or corrections, such as tests of normality and adjustment for multiple comparisons
☐	☒	A full description of the statistical parameters including central tendency (e.g. means) or other basic estimates (e.g. regression coefficient) AND variation (e.g. standard deviation) or associated estimates of uncertainty (e.g. confidence intervals)
☐	☒	For null hypothesis testing, the test statistic (e.g. F, t, r) with confidence intervals, effect sizes, degrees of freedom and P value noted<br>*Give P values as exact values whenever suitable.*
☒	☐	For Bayesian analysis, information on the choice of priors and Markov chain Monte Carlo settings
☒	☐	For hierarchical and complex designs, identification of the appropriate level for tests and full reporting of outcomes
☐	☒	Estimates of effect sizes (e.g. Cohen's d, Pearson's r), indicating how they were calculated

Our web collection on statistics for biologists contains articles on many of the points above.

Software and code

Policy information about availability of computer code

Data collection	The survey tool Limesurvey (https://www.limesurvey.org/) and the web-based annotation platform PlainSight (https://plainsight.ai/) were used to collect the reader study data.
Data analysis	All code was written in Python (3.9.9). PyTorch (1.10.0), PyTorch Lightning (1.5.10), Albumentations (1.0.3), NumPy (1.22.2), Pandas (1.4.0), SciPy (1.8.0), OpenCV (4.5.5), Scikit-learn (1.1.0), Matplotlib (3.1.1), and Seaborn (0.11.2) were used for image processing, model development and training, data analysis, and visualization.

For manuscripts utilizing custom algorithms or software that are central to the research but not yet described in published literature, software must be made available to editors and reviewers. We strongly encourage code deposition in a community repository (e.g. GitHub). See the Nature Portfolio guidelines for submitting code & software for further information.

Data

Policy information about availability of data

All manuscripts must include a data availability statement. This statement should provide the following information, where applicable:

- Accession codes, unique identifiers, or web links for publicly available datasets
- A description of any restrictions on data availability
- For clinical datasets or third party data, please ensure that the statement adheres to our policy

The dermoscopic images used to train, validate, and test our classifier are publicly available from the HAM10000 dataset (https://doi.org/10.1038/sdata.2018.161) and can be accessed here: https://dataverse.harvard.edu/dataset.xhtml?persistentId=doi:10.7910/DVN/DBW86T. The images used in our work can be filtered by

selecting the images of biopsy-verified melanoma and nevi. We used ImageNet weights to pretrain our classifier (https://pytorch.org/vision/stable/models.html). The data generated in our study, which includes the expert-annotated explanations dataset and the pseudonymized reader study data, are accessible on Figshare: https://figshare.com/s/c7feb070d066a4ccce19. Source data are provided with this paper.

Research involving human participants, their data, or biological material

Policy information about studies with human participants or human data. See also policy information about sex, gender (identity/presentation), and sexual orientation and race, ethnicity and racism.

Reporting on sex and gender	We did not collect any data on sex and gender of the clinicians participating in our reader study. The focus of the study was to investigate the influence of explainable AI on dermatologists' and dermatological residents' diagnostic behavior. We assume that gender does not play a role in this diagnostic behavior. Therefore, the exclusion of the gender aspect was a deliberate choice as we aimed to collect as little identifying personal data as possible.
Reporting on race, ethnicity, or other socially relevant groupings	We did not collect any data on race, ethnicity, or any other socially relevant groups of the clinicians participating in our reader study.
Population characteristics	116 international clinicians participated in our reader study. Among these 82 were board-certified dermatologists, 33 resident dermatologists as well as one nurse consultant specialised in dermoscopic skin cancer screening. We also collected the participating clinicians' reported experience in dermoscopy. The participants' experience was distributed in "Basis education, rare use" (n=7), "Occasional use" (n=11), "Regular use" (n=31), "Regular use and education" (n=52) and "Regular scientific discussion" (n=15). We did not collect the participants' ages but we collected the number of years they've worked as dermatologists. The distribution was "<1 year" (n=3), "1-3 years" (n=15), "3-5 years" (n=22) "5-10 years" (n=35), ">10 years" (n=41). We gave the clinicians the voluntary option to disclose the country they currently practice in. Clinicians from at least 33 countries took part in the study.
Recruitment	The participants were contacted via Email through our collaboration network and by using public contact data from the International Society for Dermoscopy website and from university clinic webpages. We also contacted participants from private clinics. As compensation, we offered them the option to be listed as a PubMed indexed collaborator on our work. Due to this line of recruitment, the reader study population is biased towards clinicians practicing in university clinics and interested in taking part in scientific research. The group of clinicians who practice dermoscopy in public hospitals and private practices is under-represented as they had less incentive to take part in the study.
Ethics oversight	The study's ethic vote is held by the University Clinic Mannheim of the Medical Faculty of the University of Heidelberg.

Note that full information on the approval of the study protocol must also be provided in the manuscript.

Field-specific reporting

Please select the one below that is the best fit for your research. If you are not sure, read the appropriate sections before making your selection.

- [x] Life sciences
- [ ] Behavioural & social sciences
- [ ] Ecological, evolutionary & environmental sciences

For a reference copy of the document with all sections, see nature.com/documents/nr-reporting-summary-flat.pdf

Life sciences study design

All studies must disclose on these points even when the disclosure is negative.

Sample size	116 clinicians participated in the reader study. In accordance with the central limit theorem, we required a minimum sample size of 30 participants; the more clinicians that participated, the better. Because our recruitment resulted in over 100 participants we opted to keep them all because more observations lead to more robust results.
Data exclusions	In each of the three reader study phases (phase 1: no AI support, phase 2: standard AI support, phase 3: explainable AI support), the clinicians were asked to diagnose 15 images of suspicious skin lesions as melanoma or nevus in a web-hosted platform. In the first phase they had to additionally select explanations and locate them on the images. They were asked to give their confidence in their diagnosis on a Likert scale from 1 to 10 in all phases, and the trust in the AI system, as well on a Liker scale, in phases 2 and 3. Prior to data collection, we registered our hypotheses, exclusion criteria and analysis plan on the Open Science Framework website, which may be accessed under https://osf.io/g3keh. We excluded the data of participants who entered constant values for trust, confidence, and/or diagnosis, such as entering a trust score of 7 for all 15 images or a diagnosis of nevus for all 15 images. We excluded images where the participant took less than 7 seconds to complete. We did not use an upper limit for time taken for exclusion because some complicated cases could take a long time to annotate. Furthermore, the participants could pause and resume working later, so a longer time taken did not necessarily imply insincere work. Images marked as having insufficient image quality (n=26) were removed for the particular participant who indicated the issue, but not for others since the image quality issues could have been related to monitor settings. As a result, we evaluated different numbers of images for each participant. None of the participants met these criteria for exclusion. Participants who dropped out in phases 2 or 3 were excluded from the study.
Replication	Replication was not performed.
Randomization	The participating clinicians were randomly allocated into 14 different groups. Participants in the same group got the same 14 distinct and one repeated images to diagnose over all three phases.

Blinding	The participating clinicians were not aware of the existence of different groups, the group they were assigned to, the repetition of images in each phase, and the distribution of melanoma and nevus images. Furthermore, they were not aware of the repeated image.

Reporting for specific materials, systems and methods

We require information from authors about some types of materials, experimental systems and methods used in many studies. Here, indicate whether each material, system or method listed is relevant to your study. If you are not sure if a list item applies to your research, read the appropriate section before selecting a response.

Materials & experimental systems

n/a	Involved in the study
☒	☐ Antibodies
☒	☐ Eukaryotic cell lines
☒	☐ Palaeontology and archaeology
☒	☐ Animals and other organisms
☒	☐ Clinical data
☒	☐ Dual use research of concern
☒	☐ Plants

Methods

n/a	Involved in the study
☒	☐ ChIP-seq
☒	☐ Flow cytometry
☒	☐ MRI-based neuroimaging
